# Substance use and addiction research in India

**DOI:** 10.4103/0019-5545.69232

**Published:** 2010-01

**Authors:** Pratima Murthy, N. Manjunatha, B. N. Subodh, Prabhat Kumar Chand, Vivek Benegal

**Affiliations:** Department of Psychiatry, De-Addiction Centre, National Institute of Mental Health and Neuro Sciences, Bangalore - 560 029, India

**Keywords:** Alcohol, drugs, India, research, substance use

## Abstract

Substance use patterns are notorious for their ability to change over time. Both licit and illicit substance use cause serious public health problems and evidence for the same is now available in our country. National level prevalence has been calculated for many substances of abuse, but regional variations are quite evident. Rapid assessment surveys have facilitated the understanding of changing patterns of use. Substance use among women and children are increasing causes of concern. Preliminary neurobiological research has focused on identifying individuals at high risk for alcohol dependence. Clinical research in the area has focused primarily on alcohol and substance related comorbidity. There is disappointingly little research on pharmacological and psychosocial interventions. Course and outcome studies emphasize the need for better follow-up in this group. While lack of a comprehensive policy has been repeatedly highlighted and various suggestions made to address the range of problems caused by substance use, much remains to be done on the ground to prevent and address these problems. It is anticipated that substance related research publications in the Indian Journal of Psychiatry will increase following the journal having acquired an ‘indexed’ status.

## INTRODUCTION

Substance use has been a topic of interest to many professionals in the area of health, particularly mental health. An area with enormous implications for public health, it has generated a substantial amount of research. In this paper we examine research in India in substance use and related disorders. Substance use includes the use of licit substances such as alcohol, tobacco, diversion of prescription drugs, as well as illicit substances.

## METHODOLOGY

For this review, we have carried out a systematic web-based review of the Indian Journal of Psychiatry (IJP). The IJP search included search of both the current and archives section and an issue-to-issue search of articles with any title pertaining to substance use. This has included original articles, reviews, case series and reports with significant implications. Letters to editor and abstracts of annual conference presentations have not been included.

Publications in other journals were accessed through a Medlar search (1992-2009) and a Pubmed search (1950-2009). Other publications related to substance use available on the websites of international and national agencies have also been reviewed. In this review, we focus mainly on publications in the IJP and have selectively reviewed the literature from other sources.

For the sake of convenience, we discuss the publications under the following areas: Epidemiology, clinical issues (diagnosis, psychopathology, comorbidity), biological studies (genetics, imaging, electrophysiology, and vulnerability), interventions and outcomes as well as community interventions and policies. There is a vast amount of literature on tobacco use and consequences in international and national journals, but this is outside the scope of this review. Tobacco is mentioned in this review of substance use to highlight that it should be remembered as the primary licit substance of abuse in our country.

## RESULTS

**Figure 1:**
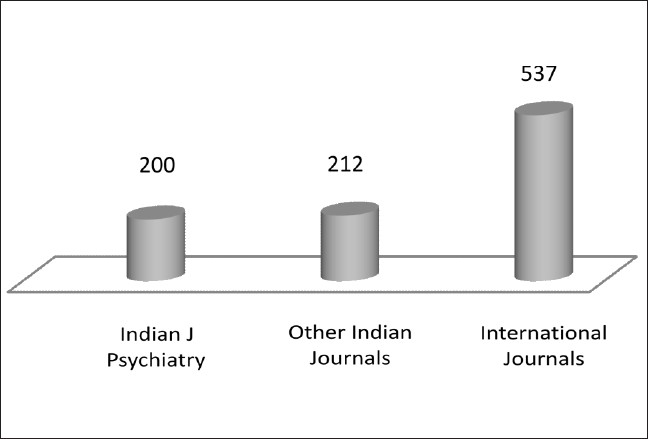
Publications in the area of substance use and related disorders

**Figure 2:**
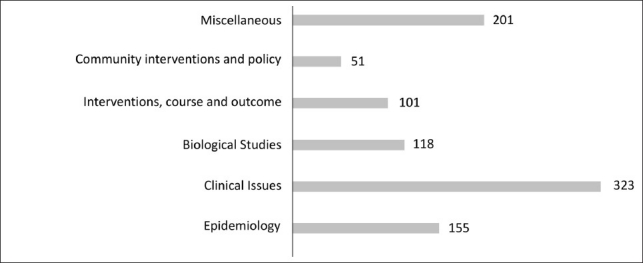
Break up of areas of publication

The number of articles (area wise) available from IJP, other Indian journals and international journals are indicated in Figures 1 and 2. A majority of the publications in international journals relate to tobacco, substance use co-morbidity and miscellaneous areas like animal studies.

## EPIDEMIOLOGY

Much of the earlier epidemiological research has been regional and it has been very difficult to draw inferences of national prevalence from these studies.

### Regional studies

Studies between 1968 until 2000 have been primarily on alcohol use [Table 1]. They have varied in terms of populations surveyed (ranged from 115 to 16,725), sampling procedures (convenient, purposive and representative), focus of enquiry (alcohol use, habitual excessive use, alcohol abuse, alcoholism, chronic alcoholism, alcohol and drug abuse and alcohol dependence), location (urban, rural or both, Slums), in the screening instruments used (survey questionnaires and schedules, semi-structured interviews, quantity frequency index, Michigan Alcohol Screening Test (MAST) etc). Alcohol ‘use/abuse’ prevalence in different regions has thus varied from 167/1000 to 370/1000; ‘alcohol addiction’ or ‘alcoholism’ or ‘chronic alcoholism’ from 2.36/1000 to 34.5/1000; alcohol and drug use/abuse from 21.4 to 28.8/1000. A meta-analysis by Reddy and Chandrashekhar[26] (1998) revealed an overall substance use prevalence of 6.9/1000 for India with urban and rural rates of 5.8 and 7.3/1000 population. The rates among men and women were 11.9 and 1.7% respectively.

**Table 1:** Regional epidemiological studies in substance use: A summary

	Year	Center	Location	Screening instrument	Population	Prevalence/1000	Focus of enquiry
Gopinath[1]	1968	Bangalore	R	Survey questionnaire	423	2.36	Alcoholism
Elnager *et al*.[2]	1971	West Bengal	R	3 stage interview	1383	13	Alcohol and drug addiction
*Dube and Handa*[3]	1971	Uttar Pradesh	R, SR and U	2 stage Interview	16725	22.8	Alcohol and drug abuse
*Varghese et al*.[4]	1973	Vellore	U	Mental health item sheet	2904	4.8	Chronic alcoholism
Thacore[5]	1975	Lucknow	U and R	Health questionnaire	2696	18.55	Habitual excessive use A-49%; C-2%
Nandi *et al*.[6]	1975	West Bengal	R	3 schedules prepared	1060	0.94	
*Lal and Singh*[7]	1978	Punjab	U	QFI	6699	293	Alcohol users
*Sethi and Trivedi*[8]	1979	Lucknow	R	Semi structured interview	2415	21.4	Drug abusers A- 43.5%; C-39.2%; O-1.4%
*Varma et al*.[9]	1980	Punjab	U and R	Structured questionnaire	1031	237	Alcohol users
Ponnudorai *et al*.[10]	1991	Madras	U	MAST	2,334	167	Alcoholism Abuse
*Premaranjan et al*.[11]	1993	Pondicherry	U	IPSS	115	34.5	Alcohol dependence
*Jena et al*.[12]	1996	Bihar	R			28.8	Alcohol/drug use
*Ghulam et al*.[13]	1996	Madhya Pradesh	U			370	Alcohol users
Singh *et al*.[14]	1998	Uttar Pradesh	U	Structured questionnaire	1806	104	Alcohol users
*Hazarika et al*.[15]	2000	Assam	R	NM	312	365	Alcohol users T-40%;A-37%; IVD-1%; IDS-1%
Sharma and Singh[16]	2001	Goa	U	RPES	4,022	1	Alcohol dependence
Mohan *et al*.[17]	2002	Delhi	U	Structured questionnaire	10,312	59	Alcohol users
*Meena et al*.[18]	2002	Haryana	U	WHO questionnaire	142000	198	Alcohol users
Silva *et al*.[19]	2003	Goa	U	AUDIT, GHQ-12	1013	211	Hazardous drinking of alcohol
Gupta *et al*.[20]	2003	Mumbai	U	Structured interview	50220	188	Alcohol users
Benegal *et al*.[21]	2003	Karnataka	U and R	Survey	21,276	153	Alcohol use
Chaturvedi *et al*.[22]	2004	Arunachal Pradesh	U	Pretested questionnaire	5,135	300	Substance abuse
Gururaj *et al*.[23]	2004	Bangalore	R, SR, Sl and U	Structured questionnaire	10,168	90	Alcohol users
Gururaj *et al*.[24]	2006	Bangalore	R, SR, Sl and U	Structured questionnaire	28,507	320	Alcohol users
*Chavan et al*.[25]	2007	Chandigarh	Sl, R	Semi structured Interview schedule	59470	69	Alcohol and drug dependence A-12%; O-0.4%; C-0.46%; N-0.13%

U - Urban; R - Rural; Sl - Slum; SR - Semi-rural; NM - Not mentioned

Regional studies between 2001 and 2007 continue to reflect this variability. Currently, the interest is to look at hazardous alcohol use. A study in southern rural India[27] showed that 14.2% of the population surveyed had hazardous alcohol use on the AUDIT. A similar study in the tertiary hospital[28] showed that 17.6% admitted patients had hazardous alcohol use.

The only incidence study on alcohol use from Delhi[17] found that annual incidence of nondependent alcohol use and dependent alcohol use among men was 3 and 2 per 1000 persons in a total cohort of 2,937 households.

### National Studies

The National Household Survey of Drug Use in the country[29] is the first systematic effort to document the nation-wide prevalence of drug use [Table 2]. Alcohol (21.4%) was the primary substance used (apart from tobacco) followed by cannabis (3.0%) and opioids (0.7%). Seventeen to 26% of alcohol users qualified for ICD 10 diagnosis of dependence, translating to an average prevalence of about 4%. There was a marked variation in alcohol use prevalence in different states of India (current use ranged from a low of 7% in the western state of Gujarat (officially under Prohibition) to 75% in the North-eastern state of Arunachal Pradesh. Tobacco use prevalence was high at 55.8% among males, with maximum use in the age group 41-50 years.

**Table 2:** Nationwide studies on substance use prevalence

Study	Sampling	Year	Number	Prevalence
National Household Survey (NHS)	Two stage probability proportional to size	2000-01	40,697 M aged 12 to 60 years in 25 states	A - 21.4%;C-3.0%;O-0.7%
National Family Health Survey (NFHS-2)	H-H	1998-99	4,86,011 aged 15-54 in 26 states	A - 17% of men and 2% women
National Family Health Survey (NFHS-3)	H-H	2005-06	124,385 F and 74,369 M aged 15-54 in 29 states	A - <1/3 of men and 2% women T - 57% men and 11% women

H-H - House to house survey; M - Male; F - Female; A - Alcohol, C - Cannabis; O - Opioids; T - Tobacco

The National Family Health Survey (NFHS)[30] provides some insights into tobacco and alcohol use. The changing trends between NFHS 2 and NFHS 3 reflect an increase in alcohol use among males since the NFHS 2, and an increase in tobacco use among women.

The Drug Abuse Monitoring System,[29] which evaluated the primary substance of abuse in inpatient treatment centres found that the major substances were alcohol (43.9%), opioids (26%) and cannabis (11.6%).

### Patterns of substance use

Rapid situation assessments (RSA) are useful to study patterns of substance use. An RSA by the UNODC in 2002[31] of 4648 drug users showed that cannabis (40%), alcohol (33%) and opioids (15%) were the major substances used. A Rapid Situation and Response Assessment (RSRA) among 5800 male drug users[32] revealed that 76% of the opioid users currently injected buprenorphine, 76% injected heroin, 70% chasing and 64% using propoxyphene. Most drug users concomitantly used alcohol (80%). According to the World Drug Report,[33] of 81,802 treatment seekers in India in 2004-2005, 61.3% reported use of opioids, 15.5% cannabis, 4.1% sedatives, 1.5% cocaine, 0.2% amphetamines and 0.9% solvents.

### Special populations

In the last decade, there has been a shift in viewing substance use and abuse as an exclusive adult male phenomenon to focusing on the problem in other populations. In the GENACIS study[34] covering a population of 2981 respondents [1517 males; 1464 females], across five districts of Karnataka, 5.9% of all female respondents (N =87) reported drinking alcohol at least once in the last 12 months, compared to 32.7% among male respondents (N = 496). Special concerns with women’s drinking include the fetal alcohol spectrum effects described with alcohol use during pregnancy.[35]

Abuse of other substances among women has largely been studied through Rapid Assessment Surveys. A survey of 1865 women drug users by 110 NGOs across the country[36] revealed that 25% currently were heroin users, 18% used dextropropoxyphene, 11% opioid containing cough syrups and 7% buprenorphine. Eighty seven per cent concomitantly used alcohol and 83% used tobacco. Twenty five per cent of respondents had lifetime history of injecting drug use and 24% had been injecting in the previous month. There are serious sexually transmitted disease risks, including HIV that women partners and drug users face.[36,37]

### Substance use in medical fraternity

As early as 1977, a drug abuse survey in Lucknow among medical students revealed that 25.1% abused a drug at least once in a month. Commonly abused drugs included minor tranquilizers, alcohol, amphetamines, bhang and non barbiturate sedatives. In a study of internees on the basis of a youth survey developed by the WHO in 1982,[38] 22.7% of males ‘indulged in alcohol abuse’ at least once in a month, 9.3% abused cannabis, followed by tranquilizers. Common reasons cited were social reasons, enjoyment, curiosity and relief from psychological stress. Most reported that it was easy to obtain drugs like marijuana and amphetamines. Substance use among medical professionals has become the subject of recent editorials.[39,40]

### Substance use among children

The Global Youth Tobacco Survey[41] in 2006 showed that 3.8% of students smoke and 11.9% currently used smokeless tobacco. Tobacco as a gateway to other drugs of abuse has been the topic of a symposium.[42]

A study of 300 street child laborers in slums of Surat in 1993[43] showed that 135 (45%) used substances. The substances used were smoking tobacco, followed by chewable tobacco, snuff, cannabis and opioids. Injecting drug use[44] is also becoming apparent among street children as are inhalants.[45]

A study in the Andamans[46] shows that onset of regular use of alcohol in late childhood and early adolescence is associated with the highest rates of consumption in adult life, compared to later onset of drinking.

### Studies in other populations

A majority of 250 rickshaw pullers interviewed in New Delhi[47] in 1986 reported using tobacco (79.2%), alcohol (54.4%), cannabis (8.0%) and opioids (0.8%). The substances reportedly helped them to be awake at night while working. In a study of prevalence of psychiatric illness in an industrial population[48] in 2007, harmful use/dependence on substances (42.83%) was the most common psychiatric condition. A study among industrial workers from Goa on hazardous alcohol use using the AUDIT and GHQ 12 estimated a prevalence of 211/1000 with hazardous drinking.[19]

### Hospital-based studies

These studies have basically described profiles of substance use among patients and include patterns of alcohol use,[49–53] opioid use,[54–56] pediatric substance use,[57] female substance use,[58] children of alcoholics[59] and geriatric substance use.[60]

Alcohol misuse has been implicated in 20% of brain injuries[61] and 60% of all injuries in the emergency room setting.[62] In a retrospective study of emergency treatment seeking in Sikkim between 2000 and 2005,[63] substance use emergencies constituted 1.16% of total psychiatric emergencies. Alcohol withdrawal was the commonest cause for reporting to the emergency (57.4%).

### Effects of substance use disorders

Mortality and morbidity due to alcohol and tobacco have been extensively reviewed elsewhere[35,64–66] and are beyond the scope of this review. The effects of cannabis have also been reviewed.[67] Mortality with injecting drug use is a serious concern with increase in crude mortality rates to 4.25 among injecting drug users compared to the general population.[68] Increased susceptibility to HIV/AIDS and other sexually transmitted diseases has been reported with alcohol[69] as well as injecting drug use.[70]

### Clinical issues

Harmful alcohol use patterns among admitted patients in general hospital has highlighted the importance of routine screening and intervention in health care settings.[71]

Peer influence is a significant factor for heroin initiation.[72] Precipitants of relapse (dysfunction, stress and life events) differ among alcohol and opioid dependents.[73] Chronologies in the development of dependence have been evaluated in alcohol dependence.[74,75]

Craving a common determinant of relapse has been shown to reduce with increase in length of period of abstinence.[76]

Alcohol dependence constitutes a significant group among the psychiatric population in the Armed Forces.[77] A study of personality factors[78] among 100 alcohol dependent persons showed significantly high neuroticism, extroversion, anxiety, depression, psychopathic deviation, stressful life events and significantly low self-esteem as compared with normal control subjects. Alcohol dependence causes impairment in set shifting, visual scanning and response inhibition abilities and relative abstinence has been found to improve this deficit.[79,80] Alcohol use has had a significant association with head injury and cognitive deficits.[81,82] Persistent drinking is associated with persisting memory deficits in head injured alcohol dependent patients.[82] Mild intellectual impairment has been demonstrated in patients with bhang and ganja dependence.[83–86]

Kumar and Dhawan[87] found that health related reasons like death/physical complications due to drug use in peers and patients themselves, knowledge of HIV and difficulties in accessing veins were the main reason for reverse transition (shift from parenteral to inhalation route).

### Evaluation and assessment

Diagnostic issues have focused on cross-system agreement[88] between ICD-10 and DSM IV, variability in diagnostic criteria across MAST, RDC, DSM and ICD[89] and suitability of MAST as a tool for detecting alcoholism.[90] The CIWA-A was found useful in monitoring alcohol withdrawal syndrome.[91]

The utility of liver functions for diagnosis of alcoholism and monitoring recovery has been demonstrated in clinical settings.[92–94] A range of hepatic dysfunction has been demonstrated through liver biopsies.[95]

A few studies have focused on scale development for motivation[96,97] and addiction related dysfunction[98] (Brief Addiction Rating Scale). An evaluation of two psychomotor tests comparing smokers and non-smokers found no differences across the two groups.[99]

Typology research has included validation of Babor’s[100] cluster A and B typologies, age of onset typology,[101] and a review on typology of alcoholism.[102]

Craving plays an important role in persistence of substance use and relapse. Frequency of craving has been shown to decrease with increase in length of abstinence among heroin dependent patients. Socio-cultural factors did not influence the subjective experience of craving.[76]

In a study of heroin dependent patients, their self-report moderately agreed with urinalysis using thin layer chromatography (TLC), gas liquid chromatography (GLC) and high performance liquid chromatography (HPLC).[103] The authors, however, recommend that all drug dependence treatment centers have facilities for drug testing in order to validate self-report.

### Comorbidity/dual diagnosis

Cannabis related psychopathology has been a favorite topic of enquiry in both retrospective[104,105] and prospective studies[106] and vulnerability to affective psychosis has been highlighted. The controversial status of a specific cannabis withdrawal syndrome and cannabis psychosis has been reviewed.[67]

High life time prevalence of co-morbidity (60%) has been demonstrated among both opioid and alcohol dependent patients.[107] In alcohol dependence, high rates of depression and cluster B personality disorders[54,108] and phobia[109] have been demonstrated, but the need to revaluate for depressive symptoms after detoxification has been highlighted.[110] It is necessary to evaluate for ADHD, particularly in early onset alcohol dependent patients.[111] Seizures are overrepresented in subjects with alcohol and merit detailed evaluation.[112] Delirium and convulsions can also complicate opioid withdrawal states.[113,114] Skin disease,[115] and sexual dysfunction[116] have also been the foci of enquiry. Phenomenological similarities between alcoholic hallucinosis and paranoid schizophrenia have been discussed.[117] Opioid users with psychopathology[118] have diverse types of psychopathology as do users of other drugs.[119]

In a study of 22 dual diagnosed schizophrenia patients, substance use disorder preceded the onset of schizophrenic illness in the majority.[120] While one study found high rates of comorbid substance use (54%) in patients with schizophrenia with comorbid substance users showing more positive symptoms[121] which remitted more rapidly in the former group,[122] other studies suggest that substance use comorbidity in schizophrenia is low, and is an important contributor to better outcome in schizophrenia in developing countries like India.[123,124]

The diagnosis and management of dual diagnosis has been reviewed in detail.[125]

### Social factors

Co-dependency has been described in spouses of alcoholics and found to correlate with the Addiction Severity scores of their husbands.[126] Coping behavior described among wives of alcoholics include avoidance, indulgence and fearful withdrawal.[127] These authors did not find any differences in personality between wives of alcoholics compared to controls.[128] Delusional jealousy and fighting behavior of substance abusers/dependents are important determinants of suicidal attempts among their spouses.[129] Parents of narcotic dependent patients, particularly mothers also show significant distress.[130]

## BIOLOGY OF ADDICTION

An understanding of the cellular and molecular mechanisms of drug dependence has led to a reformulation of the etiology of this complex disorder.[131] An understanding of specific neurotransmitter systems has led to the development of specific pharmacotherapies for these disorders.

### Cellular and molecular mechanisms

Altered alcohol metabolism due to polymorphisms in the alcohol metabolizing enzymes may influence clinical and behavioral toxicity due to alcohol. Erythrocyte aldehyde dehydrogenase was demonstrated to be suitable as a peripheral trait marker for alcohol dependence.[132] Single nucleotide polymorphism of the ALDH 2 gene has been studied in six Indian populations and provides the baseline for future studies in alcoholism.[133] An evaluation of ADH 1B and ALDH 2 gene polymorphism in alcohol dependence showed a high frequency of the ALDH2*2/*2 genotype among alcohol-dependent subjects.[134] DRD2 polymorphisms have been studied in patients with alcohol dependence, but a study in an Indian population failed to show a positive association. Genetic polymorphisms of the opioid receptor µ1 has been associated with alcohol and heroin addiction in a population from Eastern India.[135]

### Neuro-imaging and electrophysiological studies

**Figure 3:**
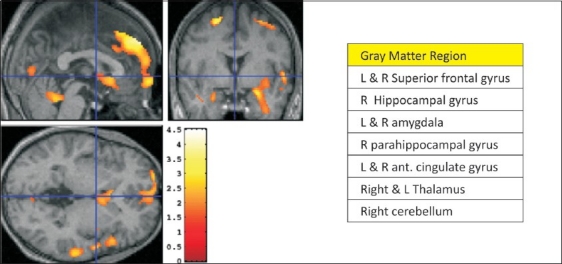
Brain volume differences between children and adolescents at high risk and low risk for alcohol dependence

Certain individuals may develop early and severe problems due to alcohol misuse and be poorly responsive to treatment. Such vulnerability has been related to individual differences in brain functioning [Figure 3]. Individuals with a high family history of alcoholism (specifically of the early-onset type, developing before 25 years of age) display a cluster of disinhibited behavioral traits, usually evident in childhood and persisting into adulthood.[136]

Early onset drinking may be influenced by delayed brain maturation. Alcohol-naïve male offspring of alcohol-dependent fathers have smaller (or slowly maturing) brain volumes compared to controls in brain areas responsible for attention, motivation, judgment and learning.[137,138] The lag is hypothesized to work through a critical function of brain maturation-perhaps delayed myelination (insulation of brain pathways).

Functionally, this is thought to create a state of central nervous system hyperexcitability or disinhibition.[139] Individuals at risk have also been shown to have specific electro-physiological characteristics such as reduced amplitude of the P300 component of the event related potential.[140,141] Auditory P300 abnormalities have also been demonstrated among opiate dependent men and their male siblings.[142]

Such brain disinhibition is manifest by a spectrum of behavioral abnormalities such as inattention (low boredom thresholds), hyperactivity, impulsivity, oppositional behaviors and conduct problems, which are apparent from childhood and persist into adulthood. These brain processes not only promote impulsive risk-taking behaviors like early experimentation with alcohol and other substances but also appear to increase the reinforcement from alcohol while reducing the subjective appreciation of the level of intoxication, thus making it more likely that these individuals are likely not only to start experimenting with alcohol use at an early age but are more likely to have repeated episodes of bingeing.[143]

## INTERVENTIONS, COURSE AND OUTCOME

Although there are a few review articles on pharmacological treatment of alcoholism,[144,145] there is a dearth of randomized studies on relapse prevention treatment in our setting.

Treatment of complications of substance use has been confined to case reports. A case report of thiamine resistant Wernicke Korsakoff Syndrome[146] successfully treated with a combination of magnesium sulphate and thiamine. Another case of subclinical psychological deterioration[147] (alcoholic dementia) improved with thiamine and vitamin B supplementation.

### Pharmacological intervention

A randomized double blind study compared the effectiveness of detoxification with either lorazepam or chlordiazepoxide among hundred alcohol dependent inpatients with simple withdrawal. Lorazepam was found to be as effective as the more traditional drug chlordiazepoxide in attenuating alcohol withdrawal symptoms as assessed using the revised Clinical Institute Withdrawal Assessment for Alcohol scale.[148] This has implications for treatment in peripheral settings where liver function tests may not be available. However, benzodiazepines must be used carefully and monitored as dependence is very common.[149]

In a study closer to the real-world situation from Mumbai, 100 patients with alcohol dependence with stable families were randomized to receive disulfiram or topiramate. At the end of nine months, though patients on topiramate had less craving, a greater proportion of patients on disulfiram were abstinent (90% vs. 56%). Patients in the disulfiram group also had a longer time to their first drink and relapse.[150] Similar studies by the same authors and with similar methodology had earlier found that disulfiram was superior to acamprosate and Naltrexone. Though the study lacked blinding, it had an impressively low (8%) dropout rate.[151,152] A chart based review has shown there was no significant difference with regard to abstinence among the patients prescribed acamprosate, naltrexone or no drugs. Although patients on acamprosate had significantly better functioning, lack of randomization and variations in base line selection parameters may have influenced these findings.[153] Short term use of disulfiram among alcohol dependence patients with smoking was not associated with decrease pulmonary function test (FEV_1_) and airway reactivity.[154]

Usefulness of clonidine for opioid detoxification has been described by various authors. These studies date back to 1980 when there was no alternative treatment for opioid dependence and clonidine emerged as the treatment of choice for detoxification in view of its anti adrenergic activity.[155–157] Sublingual buprenorphine for detoxification among these patients was reported as early as 1992. At that time the dose used was much lower, i.e. 0.6 -1.2 mg/ day which is in contrast to the current recommended dose of 6-16 mg/day. Comparison of buprenorphine (0.6-1.2 mg/ day) and clonidine (0.3-0.9 mg/day) for detoxification found no difference among treatment non completers. Maximum drop out occurred on the fifth day when withdrawal symptoms were very high.[158] A 24- week outcome study of buprenorphine maintenance in opiate users showed high retention rates of 81.5%, reduction in Addiction Severity Index scores and injecting drug use. Use of slow release oral morphine for opioid maintenance has also been reported.[159] Effectiveness of baclofen in reducing withdrawal symptoms among three patients with solvent dependence is reported.[160]

### Psychosocial

Psychoeducational groups have been found to facilitate recovery in alcohol and drug dependence.[161] Family intervention therapy in addition to pharmacotherapy was shown to reduce the severity of alcohol intake and improve the motivation to stop alcohol in a case-control design study.[162] Several community based models of care have been developed with encouraging results.[163]

### Course and outcome

An evaluation after five years, of 800 patients with alcohol dependence treated at a de-addiction center, found that 63% had not utilized treatment services beyond one month emphasizing the need to retain patients in follow-up.[164]

In a follow-up study on patients with alcohol dependence, higher income and longer duration of in-patient treatment were found to positively correlate with improved outcome at three month follow up. Outcome data was available for 52% patients; 81% of those maintained abstinence.[165] Maximum attrition was between three to six months. In a similar study among in-patients, 46% were abstinent. The drop out rate was 10% at the end of one year.[101] Studies done in the community setting have shown the effectiveness of continued care in predicting better outcome in alcohol dependence. In one study the patient group from a low socio-economic status who received weekly follow up or home visit at a clinic located within the slum showed improvement at the end of month 3, 6 and 9, and one year, in comparison with a control group that received no active follow-up intervention.[166] In a one-year prospective study of outcome following de-addiction treatment, poor outcome was associated with higher psychosocial problems, family history of alcoholism and more follow-up with mental health services.[167]

## COMMUNITY INTERVENTIONS AND POLICIES

The camp approach for treatment of alcohol dependence was popularized by the TTK hospital camp approach at Manjakkudi in Tamil Nadu.[168] Treatment of alcohol and drug abuse in a camp setting as a model of drug de-addiction in the community through a 10 day camp treatment was found to have good retention rates and favorable outcome at six months.

Community perceptions of substance related problems are useful to understand for policy development. In a 1981 study in urban and rural Punjab of 1031 respondents, 45% felt people could not drink without producing bad effects on their health, 26.2% felt they could have one or two drinks per month without affecting their health. About one third felt it was alright to have one or two drinks on an occasion. 16.9% felt it was normal to drink ‘none at all’. Alcoholics were identified by behavior such as being dead drunk, drinking too much, having arguments and fights and creating public nuisance. Current users gave the most permissive responses and non-users the most restrictive responses regarding the norms for drinking.[169] The influence of cultural norms[170] has led the tendency to view drugs as ‘good’ and ‘bad’.

Simulations done in India have demonstrated that implementing a nationwide legal drinking age of 21 years in India, can achieve about 50-60 % of the alcohol consumption reducing effects compared to prohibition.[171] However, recently there are attempts to increase the permissible legal alcohol limit. This kind of contrarian approach does not make for coherent policy.

It has been argued that the 1970s saw an overzealous implementation of a simplistic model of supply and demand.[171] A presidential address[172] in 1991 emphasized the need for a multipronged approach to addressing alcohol-related problems. Existing programs have been identified as being patchy, poorly co-ordinated and poorly funded. Primary, secondary and tertiary approaches were discussed. The address highlighted the need for supply and demand side measures to address this significant public health problem. It highlighted the political and financial power of the alcohol industry and the social ambivalence to drinking. More recently, the need to have interventions for harmful and hazardous use, the need to develop evidence based combinations of pharmacotherapy and psychosocial interventions and stepped care solutions have been highlighted.[173] Standard treatment guidelines for alcohol and other drug use disorders have suggested specific measures at the primary, secondary and tertiary health care level, including at the solo physician level.[174] An earlier report in 1988 on training general practitioners on management of alcohol related problems[175] suggests that their involvement in alcohol and health education was modest, involvement in control and regulatory activities minimal, and they perceived no role in the development of a health and alcohol policy.

There have been reviews of the National Master Plan 1994, which envisaged different responsibilities for the Ministries of Health and the Ministry of Welfare (presently Social Justice and Empowerment) and the Drug Dependence Program 1996.[176,177] A proposal for adoption of a specialty section on addiction medicine[178] includes the development of a dedicated webpage, co-ordinated CMEs, commissioning of position papers, promoting demand reduction strategies and developing a national registry.

## SUMMARY AND CONCLUSIONS

While epidemiological research has now provided us with figures for national-level prevalence, it would be prudent to recognize that there are regional differences in substance use prevalence and patterns. It is also prudent to recognize the dynamic nature of substance use. There is thus a need for periodic national surveys to determine changing prevalence and incidence of substance use. Substance use is associated with significant mortality and morbidity. Substance use among women and children is increasingly becoming the focus of attention and merits further research. Pharmaceutical drug abuse and inhalant use are serious concerns. For illicit drug use, rapid assessment surveys have provided insights into patterns and required responses. Drug related emergencies have not been adequately studied in the Indian context.

Biological research has focused on two broad areas, neurobiology of vulnerability and a few studies on molecular genetics. There is a great need for translation research based on the wider body of basic and animal research in the area.

Clinical research has primarily focused on alcohol. An area which has received relatively more attention in substance related comorbidity. There is very little research on development and adaptation of standardized tools for assessment and monitoring, and a few family studies. Ironically, though several evidence based treatments have now become available in the country, there are very few studies examining the utilization and effectiveness of these treatments, given that most treatment is presently unsubsidized and dependent on out of pocket expenditure. Both pharmacological and psychosocial interventions have disappointingly attracted little research. Course and outcome studies emphasize the need for better follow-up in this group.

While a considerable number of publications have lamented the lack of a coherent policy, the need for human resource enhancement and professional training and recommended a stepped-care multipronged approach, much remains to be done on the ground.

Finally, publication interest in the Indian Journal of Psychiatry in the area of substance use will undoubtedly increase, with the journal having become indexed.
